# Cancer Stem Cells and Their Vesicles, Together with Other Stem and Non-Stem Cells, Govern Critical Cancer Processes: Perspectives for Medical Development

**DOI:** 10.3390/ijms23020625

**Published:** 2022-01-06

**Authors:** Jacopo Meldolesi

**Affiliations:** 1 Division of Neuroscience, Scientific Institute San Raffaele, Vita-Salute San Raffaele University, via Olgnettina 58, 20132 Milan, Italy; meldolesi.jacopo@hsr.it; 2 San Raffaele Institute, Vita-Salute San Raffaele University, via Olgettina 58, 20132 Milan, Italy

**Keywords:** cancer stem cells, normal, non-stem cancer cells, mesenchymal stem cells, extracellular vesicles, niches, tumoral microenvironment, cancer differentiation, cancer progression and relapse, metastasis, therapy, clinical medicine

## Abstract

Stem cells, identified several decades ago, started to attract interest at the end of the nineties when families of mesenchymal stem cells (MSCs), concentrated in the stroma of most organs, were found to participate in the therapy of many diseases. In cancer, however, stem cells of high importance are specific to another family, the cancer stem cells (CSCs). This comprehensive review is focused on the role and the mechanisms of CSCs and of their specific extracellular vesicles (EVs), which are composed of both exosomes and ectosomes. Compared to non-stem (normal) cancer cells, CSCs exist in small populations that are preferentially distributed to the niches, such as minor specific tissue sites corresponding to the stroma of non-cancer tissues. At niches and marginal sites of other cancer masses, the tissue exhibits peculiar properties that are typical of the tumor microenvironment (TME) of cancers. The extracellular matrix (ECM) includes components different from non-cancer tissues. CSCs and their EVs, in addition to effects analogous to those of MSCs/EVs, participate in processes of key importance, specific to cancer: generation of distinct cell subtypes, proliferation, differentiation, progression, formation of metastases, immune and therapy resistance, cancer relapse. Many of these, and other, effects require CSC cooperation with surrounding cells, especially MSCs. Filtered non-cancer cells, especially macrophages and fibroblasts, contribute to collaborative cancer transition/integration processes. Therapy developments are mentioned as ongoing preclinical initiatives. The preliminary state of clinical medicine is presented in terms of both industrial development and future treatments. The latter will be administered to specific patients together with known drugs, with the aim of eradicating their tumor growth and metastases.

## 1. Introduction

Stem cells are small sub-populations of cell families, generated and concentrated at the small stroma portions that are present in the tissues of all organs of animals and humans. The most relevant of these families are the mesenchymal stem cells (MSCs), first recognized several decades ago; however, they have been of limited interest for a long time. After 1990, interest in these cells increased progressively, starting when MSCs were shown to participate in unexpected, relevant functions, including tissue regeneration and therapy for diseases [1,2]. Among the first recognized therapies were those for diseases of bone and cartilage [1,2], then those of blood, heart, brain, liver, kidney, lung, and almost all other organs [3,4,5,6,7]. The multiplicity of the discovered effects was show to depend on the limited heterogeneity of MSCs from various organs. Their mechanisms of action, based on their paracrine fusion to target cells, were first shown to be reinforced by the cooperation of soluble bioactive factors, such as cytokines and growth factors. Soon thereafter, however, the reinforcement of MSCs was found to be even larger and dependent on the secretion of their extracellular vesicles (EVs), which included two types, the exosomes and ectosomes. In order to emphasize their origin from MSCs these EVs are often called MSC-EVs. When the latter EVs were investigated separately, they were found to recapitulate most of the therapeutic effects induced by their MSCs of origin. From 2000 to now, thousands of MSC and MSC-EV studies have appeared in the literature that are focused on various aspects of their effects against diseases, supporting future perspectives for their possible development in clinical medicine [3,4,5,6,7].

In view of their limited heterogeneity, MSCs and MSC-EVs are often considered as the only stem cells and their secreted vesicles, respectively. Such definitions, however, appear short to other major classes of cells and vesicles, such as those of cancer. Many cancers express MSCs and MSC-EVs. These, on the one hand, are analogous, and on the other hand are more variable compared to those of non-cancer cells. In addition, cancer cells express a second class of stem cells, cancer stem cells (CSCs), discovered about 10 years after MSCs. A specific study about a human acute myeloid leukemia, published in 1997 [8], illustrated the small expression of extensively proliferative and self-renewing cells in the severe combined immunodefinite diseases (SCID) leukemia-initiating cells (SL-ICs), responsible for the maintenance of tumor clones [8]. Subsequent studies demonstrated that the properties attributed to SL-ICs also belong to solid cancers, such as brain and breast cancers [9,10]. Further studies revealed that small subpopulations from human brain tumors expressed the same self-renewal and exact recapitulation of the original tumor [11], maintaining, however, differentiation properties analogous to MSCs [12]. Upon their isolation, such tumor cells could be serially transplanted, thus generating protein phenocopies of the original tumor (Table 1). According to all these results, CSCs were first interpreted as the basis of solid brain tumors [11]. Subsequent studies extended this hypothesis to other types of cancers, confirming the validity of CSCs as a working model and identifying some of their highly robust surface markers, which are appropriate for the specific isolation of CSCs (see [13,14] for examples).

**Figure 1:**
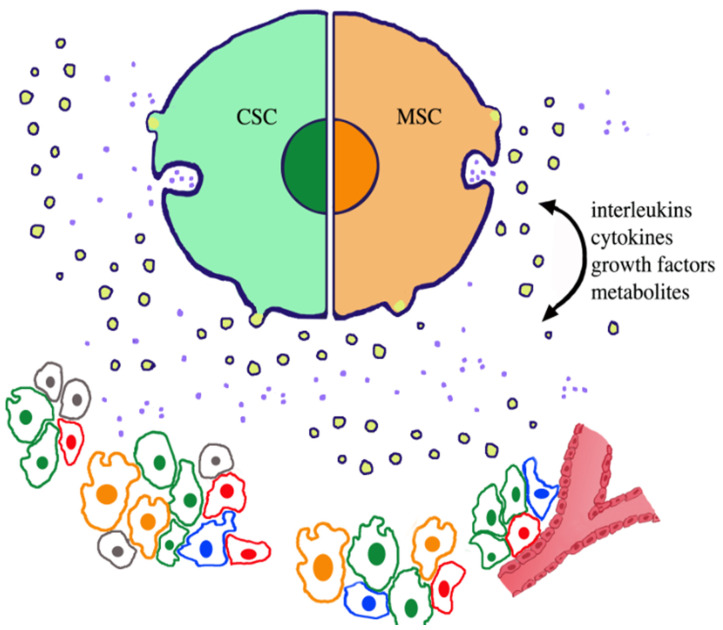
Various types of cells and their secreted vesicles that are present and active at cancer niches. At the top, two stem cells of a large size are shown attached to each other. Both the green cancer stem cells (CSC, **left**) and the orange mesenchymal stem cells (MSC, **right**) illustrate the secretion of two types of extracellular vesicles (EVs): small exosomes (light blue dots), diffusing out upon the exocytosis of their intracellular containers, the endocytic vacuoles multivesicular bodies (MVBs), and large ectosomes (membranes around yellow lumena), released by shedding of surface mini expansions. EV secretion occurs not only from the large-size cell images, but also from small cells distributed at the bottom, where, however, secretion is not shown. Secreted EVs navigate in the space among the cells. In the lower group, the cells with green and orange nuclei are CSCs and MSCs; cells with red nuclei are normal cancer cells; and cells with blue nuclei are non-cancer cells, for example immune cells that participate in cancer functions. Among their active functions, EVs mediate various types of paracrine and autocrine fusions, which are established with target cells of and outside the niches. Analogously, released fluid agents (interleukins, cytokines, growth factors) and metabolites move in all directions in the space, as suggested by the arrows. The red vessel to the right documents the process of angiogenesis, the generation of new vessels in the depth of cancers. The key role of CSCs in many processes governing cancer life depends also on their cooperation and signal exchange with the other cell types distributed within the niches.

**Figure 2:**
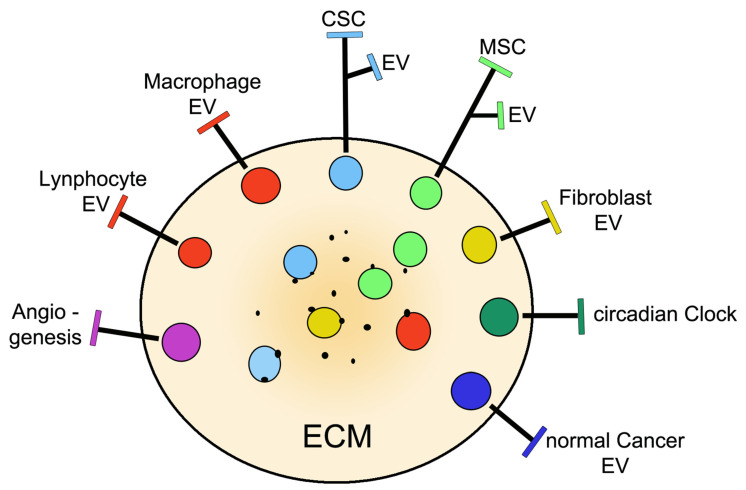
Drawing of a tumor microenvironment (TME) with reference to various types of cells, their processes, and their functions. The extracellular matrix (ECM) documents properties of the cancer environment, which is important for direct interaction with the cells. The apparent connection between the images close to the external line and their names, permits one to distinguish the nature of the present cells. The two stem cells, cancer (CSC, light blue) and mesenchymal (MSC, green), are located at the top and top right. Among the other cells, normal cancer cells, which are highly abundant in their masses (not shown), are known to differ considerably from CSCs. Fibroblasts, macrophages, and lymphocytes are filtered cells that contribute to the TME activity by their cooperation in cancer-protective and cancer-associated processes. All cells produce, by secretion, their extracellular vesicles (EVs). The highly functional and molecular relevance of the vesicles from the two stem cells are suggested by the close location of their EV to the name of their cell of origin. Cells mentioned so far, labeled by the same colors, are present also in the central area of the image. The dots at the surface of the cells are EVs in continuity, possibly involved in release or fusion, which are the processes of molecular transfer between cells. The terms angiogenesis and circadian clock do not describe single cells, but instead are complex processes taking place in TMEs. They require the cooperation of CSCs with various cooperating cells.

More recent studies, which started approximately two decades after CSC identification, led to functional and pathological characterization of CSCs cells. Small populations of heterogeneous CSCs revealed their capacity for self-renewal and aberrant differentiation to be specific for immortality and divergent lineages of cancer cells [18]. In cancer tissue CSC concentrations occur within small volumes, the niches, analogous to the stromas of non-cancer cells [19]. Niches (Figure 1) are the sites of high-degree CSC plasticity, which are dependent on transitions from slowly cycling quiescent phases to actively proliferating phenotypes, with intense secretion of their EVs [19,26]. Within niches, some of the general properties of stem cells and their vesicles depend on the cooperation of CSCs with MSCs [27,28]. Stem cells and their EVs are not alone but are accompanied by other cells and EVs: non-stem cancer cells (referred to in this review as normal cancer cells), fibroblasts, immune cells (macrophages, lymphocytes, and others) and other cells, are all involved as cooperators in cancer function [24,29,30,31,32] (Table 1). The tumor microenvironment (TME) (Figure 2), which is different from the non-cancer microenvironment, coincides in space with niches and other areas of cancer development and growth [24,25]. Finally, studies about CSCs and their associated cells/structures have been developed in areas of potential interest for therapeutic development [15,16,17]. Moreover, some of these studies are close to reaching, or have already reached, the field of clinical medicine [33].

The previous paragraphs provide a general introduction to this review. Starting with my experience of MSCs and MSC-EVs [7], interest has been focused on the expansive relevance of CSCs and their EVs. So far, most published reviews and articles in the field have been focused on single or a few specific cancers and their peculiar properties. My aim is to present a comprehensive review of cancer stem cell developments and progress within this field, focusing particularly on recently published studies and their interpretation.

## 2. CSCs and Their EVs Are Essential for Cancer Initiation and Its Processes

CSCs are small subpopulations of stem cells, which contribute to specific critical processes. In view of their considerable heterogeneity among various cancers, the effects of these cells and their EVs are not homogeneous. Most often, however, they can be envisaged according to general criteria. CSCs share some properties with MSCs, including broad proliferation and activation of various signaling processes [18,19,20,34]. Relevant to CSCs are the cellular cross-talks also involving, together with stem cells, non-cancer cells accumulated at the niches [15,16,24,25,35] (Table 1; Figure 1). The investigation of such a process is an attractive way of identifying the properties and vulnerability of most cancers [15,35].

A critical function of CSCs is immune surveillance, by which many cancers are protected by resistance to immunotherapy [24,30,32] and also to drugs [36]. Additional cellular processes that contribute to CSC function, i.e., autophagy and EV secretion, are important also for cell survival [21,37,38,39,40]. Autophagy, which contributes to the traffic, turnover, and secretion of cytoplasmic proteins and membranes, favors CSCs and their vesicles [37,38]. Inhibitors, which affect EV secretion, are now believed to be highly important and attractive for anticancer therapy [37,39]. Bypassing autophagy inhibition can be achieved by drugs [39], thanks to the acute adaptability and plasticity of CSCs [17]. EVs duplicate many effects of CSCs and are essential for the communication between various types of cells concentrated in the niches [7,21,28].

However, most key properties of CSCs are specific [17,34]; their modulation appears largely focused on self-renewal and multi-lineage differentiations [20,21], which is different from those of the MSC family. CSCs show multi-lineage properties, leading to the generation of distinct cancer subtypes [18,21]. Moreover, CSCs participate in many critical processes of cancer, from initiation and progression to formation of metastases, therapy resistance, and cancer relapse (Table 1) [17,21,22,23]. Based on their unique role in most critical processes, CSCs are now recognized as possible key targets of anti-cancer therapy.

For the origin of CSCs, proliferation is predominant and induces many effects reported in Table 1. The relevance of some differentiation has also been proposed. In normal cancer cells, such a possibility has been confirmed by experiments showing that, upon their elimination, CSCs are replaced by surrounding normal cancer cells via the acquisition of specific properties [20,21]. The sites of CSC differentiation are distributed at the niches, which are present in all cancers [19,35]. The function of CSCs depend on their secretion of both soluble factors (interleukins, cytokines, growth factors) and EVs [21,27,28]. Concomitantly, CSCs receive soluble signaling factors together with EVs that are released by normal cancer and other niche cells [19]. Figure 1 illustrates the general structure and analogous functions of a single CSC and MSC shown in a large format. Below the two stem cells is a list of the various cells coexisting in the niche, with their bidirectional exchange of signals. With their collaboration with MSCs, normal cancer cells and non-stem/non cancer cells are essential for many specific CSC activities [20,29,30,31,32,35,40].

Important interactions of CSCs, however, are not direct but occur via their secreted EVs [21,27,28,36,41]. Up until now, a clear distinction between the vesicles secreted by CSCs and MSCs has not been reported. Therefore, the CSC-dependent vesicles are simply indicated as EVs, and are abundant within niches. Whenever possible, the molecular profiling of circulating EVs provides a non-invasive but promising means of diagnosing cancer, by monitoring its state and predicting its expected development [27,28]. In some cases, however, due to the similarities and frequent co-localization of the two EV types, their distinctions remain unclear. It should be emphasized, however, that studies have been reported showing that EVs from CSCs contribute significantly to tumor progression. Among the processes involved are tumor resistance, metastasis, angiogenesis, and maintenance of stemness and immune suppression [21,23,36,41,42,43]. The main role of EVs also includes their fusion with macrophages and other immune cells [30,31]. CSCs and their EVs are able to modulate cancer cell proliferation by the release of proteins [44,45,46] and miRNAs [30,47,48,49,50,51]. Examples are increasing, with results in favor or against cancer. miRNAs from CSC/EV have been reported recently. The first of these are in favor. The miR-92/PD-L1 pathway contributes to the suppression of immune cell function [30] and miR-200c stimulates the metastatic traits in colorectal cancer [47]. On the other hand, human liver cancer is attenuated in vitro and in vivo by miR-145 and miR-200 [48]; inhibition of lung metastasis is induced in osteosarcoma by miR-101 [49]; miR-8063 inhibits self renewal of GSC [50]; miR-663 inhibits the CSCs of a glioma [51]; and miR-1468-5p promotes a tumor immune escape [52]. Interestingly, many of the effects reported in this section, induced by CSCs and miRs, are mediated by the activation of various signaling pathways such AKT [22,46], GSK3-β [22], Wnt/β-catenin [22,23,50,51], and TGF-β/SMAD [53].

## 3. Role of MSCs and MSC-EVs

MSCs and MSC-EVs are concentrated within niches together with CSCs, other EVs, normal cancer cells, and non-cancer cells involved in specific processes of cancer relevance (Figure 1). The role of MSCs/MSC-EVs in the development of cancer, already reported during the last decade (see for example [27,29,54,55,56,57]), has been confirmed recently by meta-analyses and critical interpretations [58,59]. The different role, positive or negative, of mesenchymal stem components could be due to their heterogeneity or, alternatively, to the state of the cancers involved, dependent on their CSCs and EVs [56,57]. Results reported here often appear due to the cooperation of the two stem-cell types and their EVs, with the induction of therapy forms such as those against pancreatic and colon cancers [60,61]. In contrast, the non-small cell lung cancer appears reinforced [17]. Interestingly, the positive results induced by MSC in some tumors were not confirmed in their metastases where MSC responses were negative [15,34]. In this case, therefore, different mechanisms taking place in different areas of cancer pathology, appear to govern an apparently single process.

The cancer role of MSCs has also been investigated via molecules (proteins, lipids, miRNAs), most often released from the EV cargoes. Identification of miRNAs active at one or more steps of MSC signaling cascades [59,60,61,62], and which are different from those involved in CSC action [47,48,49,50,51] were already established some years ago. Recent findings have led to the identification not only of miRs but also of signaling cascades they contribute to in order to activate. Specifically, miR-193a has been found to impede proliferation, migration, and invasion of its colon cancer cells via down-regulation of the FAK kinase [60]; miR-133b suppresses glioma cell progression via activation of the Wnt/β- catenin signaling cascade [63]; and overexpressed miR-34a, active on breast cancers, induces repression of their cell proliferation and growth [64]. Analogously, miR-199, via stimulation of the mTOR pathway, increases the chemo-sensitivity of hepatocellular carcinoma cells [65]. In contrast, miR-208a induces proliferation, migration, and invasion of osteosarcoma cells [66] and two miRNAs, miR-21-5p and miR-130b-3p, promote the growth and mobility of two types of lung cancer by regulating their FoxO3 axis [67,68].

The MSC-EV actions reported so far refer to modulations of cancer actions analogous, but not identical, to those operating in non-cancer organs. This, however, is not the only function of vesicles. In addition to their release of molecules, EVs operate by fusing with various types of cells: CSCs, normal cancer, and non-cancer cells [27,28]. Further functions of MSC-EVs include processes, such as invasiveness, immunology, and angiogenesis, which are presented in the next TME Section.

## 4. The Tumor Microenvironment: The Role of CSCs and Cooperative Cells

A TME, a dynamic milieu of stem and other types of cells, corresponds to the peculiar heterogeneous environment existing within niches. In addition, a TME is distributed in the spaces surrounding tumor masses [69,70]. For the development of cancer processes, such as migration and invasion, resistance to antitumor treatments, proliferation, and growth of metastases, CSCs are required to operate in the specific environment of TMEs, their immune agents, and other components [24,71,72].

TMEs include components of the extracellular matrix (ECM) together with either type of stem cell, normal cancer cells and infiltrating non-tumor cells, which are all involved in relevant processes (Figure 2). These components govern various peculiar aspects of cancer volumes. The ECM components, which are different from the corresponding matrices of healthy tissues, make the various basic properties of the environment possible, including the pH and various ions together with specific cancer markers, which is of interest for their binding to cell surfaces [71,72]. EVs, secreted by all cell types present in a TME, operate together with soluble agents such as interleukins, cytokines, growth factors, and various metabolites [72,73]. The interactions among environmental cells, mediated by their EVs, result in their paracrine and autocrine fusions, thereby participating in the modulation of cancer progression [19,67].

Recent studies have clarified processes by which cancer and non-cancer stem cells, during their navigation in the TME and upon their crosstalk, stimulate cancer development. An important contribution depends on the inhibition of immune cells by the so-called immune escape process [52,69]. Tumor-initiating CSCs shape their microenvironment into immunosuppressive barriers and pro-tumorigenic niches, all including filtered immune cells, which are most often macrophages and lymphocytes [69,70]. The interactions between macrophages and CSCs contribute to the development, association, and dissemination of tumor-associated cells dependent on the signaling of miRNAs released from EV cargoes [31,74]. A few processes, promoted by the miR-1468-5p, induce the immune escape of tumors via the immunosuppressive reprogramming of lymphatic vessels [52]. B cell proliferation can be inhibited by EVs secreted by CSCs, which is an approach of future therapeutic interest [75]. Additional immunosuppressive processes have been reported that occur in the TME: suppression of immune cell function induced by EVs secreted by cancer-associated fibroblasts, with ensuing stimulation of cancer progression [30]; and responses apparently triggered by miR-146a that induces transition from MSCs into cancer-associated fibroblasts [76] (Figure 2).

In addition to their transition processes already mentioned [68,69,70,71,72,73,74,75,76], TME governs angiogenesis (Figure 1 and Figure 2), a process of tumor growth by which new blood vessels develop from pre-existing ones. Angiogenesis depends on cancer EVs cooperation via their miRNAs and positive factors (vascular endothelial growth factor and matrix metalloproteinases), together with the suppression of another factor inhibiting the hypoxia-inducible factor [77]. The ensuing effect is an activation of angiogenic signaling pathways in normal endothelial cells, with ensuing formation of new cancer vessels, where endothelial cells are differentiated from CSCs [25,77,78]. Immunoblocking of angiogenesis, a sedative process of cancer progression, could be prevented in TMEsby the crosstalk of stem cells with immune cells [79]. Analogously, the inhibition of angiogenesis by the transcription repressor FoxO1 can be prevented by the miR-135b of EVs from endothelial cells [80].

A process regulated by a TME is the circadian clock (Figure 2). The connection established here is due to the high degree of CSC property and its functional plasticity (Table 1), which is dependent on the transition from slowly cycling quiescent phases to actively proliferating phenotypes [19,26]. Circadian clocks contribute to cancer growth by regulating stem cells and the TME. Among TME processes, the circadian clock operates on immune escape and angiogenesis. The effect of the circadian clock on tumor progression is probably dependent on its effects on stem cells and the pro-tumor TME [81,82].

## 5. Development of Therapies: Methods and Tools

The present section, based on the stem-cell properties presented so far about cancers and the mechanisms of their development, is focused on the numerous anti-cancer processes. The interest in stem cells started from their possible development in clinical practice [3,4]. In most cases, however, such developments did not occur, thus knowledge about these studies, which is quite variable in nature and mechanisms of action, remained at preclinical levels. The aim of this section is to summarize the present state of the field of therapy.

The processes to be considered that deal with the specificity of the miRs that modulate the CSC gene expression [83] and the cascades involved in their signaling, are numerous [41,53,64,84]. The opposite effects of inhibition or reinforcement are induced by drugs, rather than CSC action [36,84]. Additional promising perspectives have been identified recently. Details regarding the methods and tools employed in the procedures mentioned from now on can be found in the relative publications. Among such procedures are the role of autophagy in the CSC cancer development [44,45]; the reductions in CSC levels induced by changes of their metabolism [85]; the dependence on CSCs together with MSCs and EVs for cancer distribution to various organs [16,58,86]; and the relevance of therapy for two types of processes: the immune escapes [24,32] and the epithelial-mesenchymal and MSC-fibroblasts transitions [31,51,76]. From the multiplicity of these approaches it is expected that specific therapeutic processes, aimed to eradicate tumors by preventing their main processes, such as metastases, tumor relapses, and drug resistance, can be induced by specific effects, such as reverse responses and increased drug efficacy [35,36,47]. In conclusion CSCs, MSCs, and their EVs can be considered as promising tools for the treatment of cancers and disorders, aimed to overcome the limitations, such as low efficacy and toxicity, of ongoing cell therapies.

Any strategy against CSCs depends on the efficacy of its cancer therapy. With time, many pharmacological approaches have been established, affecting critical properties of their target cells [44,56,64]. However, only a few conventional therapies have been fully successful. Improved results have been obtained by the combination of distinct treatments. Nanomedicines [17], which started over 10 years ago, have reinforced therapy by accurate combinatorial approaches, i.e., by drugs and genes, qualified by targeting and combinational deliveries. By such an approach, the poor prognosis observed in patients with various types of cancers, treated by potentially appropriate, but lunefficient conventional drugs, is now greatly improved [17,40]. More integrated nanomedicine approaches have been applied against metastatic prostate and other cancers, based on conventional drugs combined with drugs of different types, such as docetaxel, a cytotoxic agent that disrupts microtubule formation and thus halts cell division; *meta*-tetrahydroxyphenyl chlorin, a photodynamic drug employed for treatment of peritoneal metastases from carcinomatosis; or the chemotherapeutic agent doxorubicin, integrated into lipid bubbles [87,88,89]. Analogous successes have been obtained by the same doxorubicin delivered, however, within MSC-EVs [90]. The drugs employed in both nanomedicine and EVs are encapsulated by an engineered technique governed by a GMP technique [33,90]. Such encapsulation succeeds to target cancers with superior selectivity, thus obtaining therapeutic effects that are much stronger than those of free drugs [17,86,87,88,89,90,91,92,93]. In conclusion, the introduction of nanomedicine is interesting, however its medical employment is still under development.

Cancer therapy, such as the one summarized here, has been recently reconsidered from the point of view of CSCs, the stem cells that have gained special attention as avenues of intervention. The interactions of CSCs with surrounding cells and their EVs are critical, operating via several mechanisms including fusions to target cells with the ensuing release of critical cargo molecules, such as proteins and miRNAs [83]. The innovative understanding of cancer based on specialized stem cells has contributed to new benefits, oriented to more efficient therapeutic treatments. The intense investigations that are ongoing at present are expected to develop into efficient therapies in the next few years [94].

## 6. Progress in Clinical Medicine

As already mentioned in the previous section, the intense cancer CSC studies carried out during the last several years are aimed at the conversion of basic knowledge to clinical trials and medical employment. However, CSC-target therapy is affected by CSC heterogeneity. Therefore, more in-depth knowledge and technology are still required to develop novel therapies. Moreover, novel strategies are needed to effectively eradicate both tumor growth and metastasis, while taking into account the TME, which plays a key role in tumor-cell plasticity [95]. At present, studies involving the engineering of stem cell therapies may ultimately induce the development of new agents employed for clinical practice. The sites specific for cancer growth may be useful for initial clinical trials that remain to be developed and evaluated [96]. While research of CSCs has exploded during the last few years, its development towards clinical practice is still at a preliminary stage [36,97]. The present state of the problem will be considered from two points of view concerning the operative properties of CSC and their EVs that are needed for the entrance into clinical practice and the clinical therapy needed for various diseases.

Biotechnological and pharmaceutical companies are considering, with interest, the chance to invest in clinical practice by manufacturing, safety, and efficacy of their products. For this, they intend to establish sources of cancer cell types and analyze their diseases. It will be important to identify animal models that are appropriate for the initial experiments and that are essential for future studies based on toxicities and pharmacological investigations [95]. The manufacturing of molecules will then be started, also keeping in mind the number of potential patients considered. In view of the critical role of CSCs, targets of possible cancers will be based on these cells and their secreted EVs. Upon their characterization, such tools will be purified and then analyzed by pharmacological tools before the start of their production [97,98].

A form of medical practice, often employed for non-cancer diseases, is regenerative medicine, which is based on affected tissues including the brain, the blood vessels, and other organs (for details see [75,99]). In case of cancers, this approach is employed, however not frequently [88,96]. In the field of cancer, ongoing research is about personalized medicine [12,20,100]. Cancer EVs associated to a drug delivery system have already reached early phases in clinical trials [97,101]; however, the clinical application of this is still far away. Protocols for EV loading, modification, and isolation need to be standardized for large-scale production. Careful evaluation of the findings concerning qualification, characterization, and production of the methods employed, include pharmacokinetics, targeting and transfer of drugs to appropriate sites; assessment of safety profiles; and others [95,96,97,98,100,101,102].

## 7. Conclusions

At present, stem cells are attracting great interest, with innovative properties reported almost every month. MSCs undergo various types of differentiation that contribute to their heterogeneity. However, the properties and functions of the various forms of MSCs and their EVs, produced for research and employment in medicine, have been characterized and are not profoundly different from each other. Most forms of these stem cells are therefore called by the same nomenclature. However, MSCs participate in, but do not cover, a large case of cancers. The CSC system is, in fact, more complex than that of MSCs. The two types of coexisting stem cells, MSCs and CSCs, are able to cooperate, however, only by some of their properties. The remaining critical properties are specific. In the expression of the latter properties CSCs and their EVs predominate [19,20,21,23,27,35,43,47].

In cancer niches and TMEs (Figure 1 and Figure 2), CSCs and their EVs coexist not only with cells of the MSC family, but also with normal cancer cells and various non-cancer cells, such as fibroblasts, macrophages, and other immune cells [15,24,25,30,31,32,67,68,69,70,71,72,73,74]. Processes of key relevance for cancer, such as immune escape, drug resistance, and cancer relapse, i.e., the processes that pose the greatest barriers to cancer care, are driven by the CSC stem cell program andin some cases withthe specific participation of non-cancer cells [30,31,74,76]. In additional processes, the predominant role of CSC is also sustained by its participation in apparently independent events, such as drug efflux, involvement of the autophagic machinery, secretion of cytokines, and other factors [37,64,73]. CSCs’ heterogeneity occurs according to multi-lineage differentiation, leading to distinct cancer subtypes. The bidirectional exchange of signals between CSCs and the other cells accumulated in the niches is important for preserving the activity and the specificity of the various cells involved [20,46,51]. The role of CSCs can be envisaged from key steps of its action, such as cancer initiation and its progression up to metastasis generation, whicha re often conceived together with the collaboration of the other cells. Relevance of CSCs and their collaborators, established in the niches and TSEs, are therefore essential for cancer initiation and development [21,22,23,40,41]. The interactions among cells active in cancer has been interpreted for years according to a hierarchical concept, which is based on the general predominance of CSCs. Recently this interpretation has been questioned, based on an alternative interpretation of clonal evolution and stemness phenotype models [103,104]. Due to these proposals, the predominant CSC role should be postponed until the proximal future. CSC remains, however, as a possible target of innovative therapies developed by operational terms; innovative not only for scientific studies, but especially for clinical medicine.

## Figures and Tables

**Table 1:** History and properties of CSCs.

Discovery	1997–2004: First discovered in leukemia, then in cancers of the brain and other organs	[8,9,10,11,12,13,14].
Concentrated in:	Niches and tumor microenvironments (TMEs).	
Co-distribution and co-operation of CSCs with:	MSCs, normal. Cancer cells, fibroblasts, macrophages, other immune cells	[15,16,17].
Basic functions:	Cancer-cell initiation, immortality, self renewal, multi-lineage divergence, differentiation	[18,19,20,21].
Peculiar functions:	Progression, functional plasticity, metastases, therapy resistance, cancer relapse	[17,19,21,22,23].
Secretions:	Cytokines, interleukins, growth factors	[24,25].
Released extracellular vesicles:	Exosomes and ectosomes	[3,4,5,6,7,19,26].
Replacement (if needed):	By surrounding normal cancer cells	[20,21].

## Data Availability

The data of this review will be available to all scientists with specific interests.

## Abbreviations

CSCs	cancer stem cells
ECM	extracellular matrix
EVs	extracellular vesicles
GMP	good manufacturing practice
miRNAs, miR-number	microRNAs
MSCs	mesenchymal stem cells
MVB	multivesicular body
TME	tumoral microenvironment

## References

[1] Caplan A.I. (1991). Mesenchymal stem cells. J. Orthop. Res..

[2] Yung H.E., Ceballos E.M., Smith J.C., Mancini M.L., Wrigh R.P., Regan B.L., Bishell I., Lucas P.A. (1993). Pluripotent mesenchymal stem cells reside within avian connective tissue matrices. Vitr. Cell Dev. Biol.-Anim..

[3] Ballas C.B., Zieske S.P., Gerson S.L. (2002). Adult bone marrow stem cells for cell and gene therapies: Implications for greater use. J. Cell Biochem. Suppl..

[4] Devine S.M. (2002). Mesenchymal stem cells: Will they have a role in the clinic?. J. Cell Biochem. Suppl..

[5] Katsuda T., Kosaka N., Takeshita F., Ochiya T. (2013). The therapeutic potential of mesenchymal, stem cell-derived extracellular vesicles. Proteomics.

[6] Askenase P.W. (2021). Ancient evolutionary origin and properties of universally produced natural exosomes contribute to their therapeutic superiority compared to artificial nanoparticles. Int. J. Mol. Sci..

[7] Racchetti G., Meldolesi J. (2021). Extracellular vesicles of mesenchymal stem cells: Therapeutic properties discovered with extraordinary success. Biomedicines.

[8] Bonnet D., Dick J.E. (1997). Human acute myeloid leukemia is organized as a hierarchy that originates from a primitive hematopoietic cell. Nat. Med..

[9] Singh S.K., Clarke I.D., Terasaki M., Bonn V.E., Hawkins C., Squire J., Dirks P.B. (2003). Identification of a cancer stem cell in human brain tumors. Cancer Res..

[10] Al-Halj M., Wicha M.S., Benito-Hernandez A., Morrison S.J., Clarke M.F. (2003). Prospective identification of tumorigenic breast cancer cells. Proc. Natl. Acad. Sci. USA.

[11] Singh S.K., Hawkins C., Clarke J.D., Squire J.A., Bayani J., Hide T., Henkelman R.M., Cusimano M.D., Dirks P.D. (2004). Identification of human brain tumour initiating cells. Nature.

[12] Liu B.B., Quin L.X., Liu Y.K. (2005). Adult stem cells and cancer stem cells: Tie or tear apart?. J. Cancer Res. Clin. Oncol..

[13] Fang D., Thiennanga K.N., Leishear K., Finko R., Kulo A.N., Holz S., Van Belle P.A., Xu X., Elder D.E., Herlyn M. (2005). A tumorigenic sub-population with stem cell properties in melanoma. Cancer Res..

[14] Dalerba P., Scott J.D., Park I.K., Liu R., Wang X., Cho R.W., Hoey T., Gurney A., Huang E.H., Simeone D.M. (2005). Phenotypical characterization of human colorectal cancer stem cells. Proc. Natl. Acad. Sci. USA.

[15] Attar-Schneider O., Dabbah M., Drucker L., Gottfried M. (2020). Niche origin of mesenchymal stem cell-derived microvesicles determines opposing effects on NSCLC: Primary versus metastatic. Cell Signal..

[16] Li T., Zhou X., Wang J., Liu Z., Han S., Wan L., Sun X., Chen H. (2020). Adipose-derived mesenchymal stem cells and extracellular vesicles confer antitumor activity in preclinical treatment of breast cancer. Pharmacol. Res..

[17] Gener P., Gonzalez-Callejo P., Serra-Franzoso J., Andrade F., Rafael D., Albasolo I., Schwartz S. (2020). The potential nanomedicine to alter cancer stem-cell dynamics: The impact of extracellular vesicles. Nanomedicine.

[18] Luo H.T., Zheng Y.Y. (2021). Dissecting the multi-omic atlas of the exosomes released by human lung carcinoma stem-like cells. NPJ Genom. Med..

[19] Ye J., Wu D., Wu P., Chen Z., Huang J. (2014). The cancer stem cell niche: Cross talk between cancer, stem cells and their microenvironment. Tumor Biol..

[20] Afify S.M., Seno M. (2019). Conversion of stem cells to cancer stem cells: Undercurrent of cancer initiation. Cancers.

[21] Afify S.M., Hassan G., Yan T., Seno A., Seno M. (2021). Cancer stem cell initiation by tumor-derived extracellular vesicles. Methods in Molecular Biolog.

[22] Kim D.K., Kim Y.N., Kim Y.E., Lee S.Y., Shin M.J., Do E.K., Choi K.U., Kim S.C., Suh D.S., Kim J.H. (2021). TRIB2 stimulates cancer stem-like properties through activation of AKT-Wnt-β-catenin signaling axis. Mol. Cells.

[23] Yin H., Gao T., Xie J., Hung Z., Zhang X., Yang F., Qi W., Yang Z., Zhou T., Gao G. (2021). FUBP1 promotes colorectal cancer stemness and metastasis via DVL1-mediated activation of WNT/β-catenin signaling. Mol. Oncol..

[24] Ferguson L.P., Diaz E., Reya T. (2021). The role of the microenvironment and immune system in regulating stem cell fate in cancer. Trends Cancer.

[25] Ludwig N., Rubenich D.S., Zareba L., Siewiera J., Pieper J., Braganhol E., Reichert T.E., Szczepanski M.J. (2020). Potential role of tumor cell and stroma cell-derived small in promoting a pro-angiogenic tumor microenvironment. Cancers.

[26] Fukushi D., Takahashi R.S., Mochizuki M., Fujimori S., Kokure T., Sugai T., Iwai W., Wakui T., Abue M., Murakami K. (2021). BEX2 is required for maintaining dormant cancer stem cell in hepatocellular carcinoma. Cancer Sci..

[27] Soares Lindoso R.S., Collini F., Vieyra A. (2017). Extracellular vesicles as regulatory of tumor fate: Crosstalk among cancer stem cells, tumor cells and mesenchymal stem cells. Stem Cell Investig..

[28] Desrochers L.M., Antonyak M.A., Crione R. (2016). Extracellular vesicles: Satellites of information transfer in cancer and stem cell biology. Dev. Cell.

[29] Bajetto A., Thellung S., Dellacasagrande I., Pagano A., Barbieri F., Florio T. (2020). Cross talk between mesenchymal and glioblastoma stem cells: Communication beyond controversies. Stem Cells Transl. Med..

[30] Dou D., Ren X., Han M., Xu X., Gu Y., Wang X. (2020). Cancer-associated fibroblasts-driven exosomes suppress immune cell function in breast cancer via the miR-92/PD-L1 pathway. Front. Immunol..

[31] Aramini B., Masciale V., Grisendi G., Banchelli F., D’Amico R., Majorana A., Morandi U., Dominici M., Haider K.H. (2021). Cancer stem cells and macrophages: Molecular connections and future perspectives against cancer. Oncotarget.

[32] Castagnoli L., De Santis F., Volpari T., Vernieri C., Tagliabue E., Di Nicola M., Pupa S.M. (2020). Cancer stem cells: Devil or savior-looking behind the scenes of immunotherapy failure. Cells.

[33] Chen Y.S., Lin E.Y., Chiou T.W., Harn H.J. (2019). Exosomes in clinical trial and their production in compliance with good manufacturing practice. Ci Ji Yi Xue Za Zhi.

[34] Koshkin S.A., Anatskaya O.V., Vinogradov A.E., Uversky V.N., Dayhoff G.W., Bystriakova M.A., Pospelov V.A., Tolkunova E.N. (2021). Isolation and characterization of human colon adenocarcinoma stem-like cells based on the endogenous expression of stem markers. Int. J. Mol. Sci..

[35] Oshimori N., Guo Y., Taniguchi S. (2021). An emerging role of cellular crosstalk in the cancer stem cell niche. J. Pathol..

[36] Zhang J., Song Q., Wu M., Zheng W. (2020). Emerging roles of exosomes in the chemoresistance of hepatocellular carcinoma. Curr. Med. Chem..

[37] Raudenska M., Balvan J., Masarik M. (2021). Crosstalk between autophagy and endosome-related secretory pathways: A challenge for autophagy-based treatment of solid cancer. Mol. Cancer.

[38] Kim Y.H., Kwak M.S., Lee B., Shin J.M., Aum S., Park I.H., Lee M.G., Shin J.S. (2021). Secretory autophagy machinery and vesicular trafficking are involved in HMG1 secretion. Autophagy.

[39] Brun S., Pacussi J.M., Gifu E.P., Bestion E., Macek-Ilikova Z., Wong G., Bassissi F., Mehar S., Courcambeck J., Merle P. (2021). GNS561, a new autophagy inhibitor active against cancer stem cells in hepatocellular carcinoma and metastasis from colorectal cancer. J. Cancer.

[40] Pan T., Xu J., Zhu X. (2017). Self renewal molecular mechanisms of colorectal cancer stem cells. Int. J. Mol. Med..

[41] Lopez de Andres J., Grinan-Sison C., Jimenez G., Marchal J.A. (2020). Cancer stem cell secretome in the tumor micro-environment: A key point for an effective personalized cancer treatment. J. Hematol. Oncol..

[42] Bebelman M.P., Janssen E., Peftel D.M., Crudden C. (2021). The forces driving cancer extracellular vesicle secretion. Neoplasia.

[43] Su C., Zhang J., Yarden Y., Fu L. (2021). The key roles of cancer stem cell-derived extracellular vesicles. Signal Transduct. Target. Ther..

[44] Maruyama M., Nakano Y., Nashimura T., Ivata R., Matsuda S., Hayashi M., Nakai Y., Nonaka M., Sugimoto T. (2021). PC3-secreted microproteins expressed in glioblastoma stem-like cells and human glioma tissues. Biol. Pharm. Bull..

[45] Wang M.J., Chen J.J., Song S.H., Su J., Zhao L.H., Liu Q.G., Yang T., Chen Z., Liu C., Fu Z.R. (2021). Inhibition of SIRTI1 limits self-renewal and oncogenesis by inducing senescence of liver cancer stem cells. Hepatol. Carcinoma.

[46] Phiboonchalyanan P.P., Puthogking P., Chawlarean V., Harikarnpakdee S., Sukprasansap M., Chanvorachote P., Priprem A., Goyitraprom P. (2021). Melatonin and its derivative disrupt cancer stem-like phenotypes of lung cancer cells via AKT down-regulation. Clin. Exp. Pharmacol. Physiol..

[47] Tang D., Xu X., Ying J., Xie T., Cao C. (2020). Transfer of metastatic traits via miR-200c in extracellular vesicles derived from colorectal cancer stem cells is inhibited by atractylenolide. Clin. Transl. Med..

[48] Brossa A., Fonsato V., Grange C., Tritta S., Tappar M., Calvetti R., Cedrino M., Fallo S., Gontero P., Camussi G. (2020). Extracellular vesicles from human liver stem cell-derived tumor growth in vitro and in vivo. Int. J. Cancer.

[49] Zhang K., Dong C., Chen M., Yang T., Wang X., Gao Y., Wang L., Wen Y., Chen G., Wang X. (2020). Extracellular vesicle-mediated delivery of miR-101 inhibits lung metastasis in osteosarcoma. Theranostics.

[50] Chen Z.Q., Yuan T., Jiang H., Yang Y.Y., Wang L., Fu R.M., Luo S.Q., Zhang T., Wu Z.Y., Wen K.M. (2021). MicroRNA-8063 targets heterogeneous nuclear ribonucleoprotein AB to inhibit the self renewal of colorectal cancer cells via the Wnt/β-catenin pathway. Oncol. Rep..

[51] Alraouil N.N., Hendrayani S.F., Ghebeh H., Al-Mohanna F.H., Aboussekhra A. (2021). Osteoprotegerin (OPG) mediates the anti-carcinogenic effects of normal breast fibroblasts and target cancer stem cells via the Wnt/β-catenin pathway. Cancer Lett..

[52] Zhou C., Wei W., Ma J., Yang Y., Liang L., Zhang Y., Wang Z., Chen X., Huang L., Wang W. (2021). Cancer-secreted exosomal miR-1468-5p promotes tumor immune escape via the immunosuppressive reprogramming of lymphatic vessels. Mol. Ther..

[53] Wang L., Lang B., Zhou Y., Ma J., Hu K. (2021). Upregulation of the miR-663a inhibits the cancer stem cell-like properties of glioma via repressing the KDM2A-mediated TGF-β/SMAD signaling pathway. Cell Cycle.

[54] Zhu W., Huang L., Li Y., Zhang X., Gu J., Yan Y., Xu X., Wang M., Qian H., Xu W. (2012). Exosomes derived from human bone marrow mesenchymal stem cells promote tumor growth in vivo. Cancer Lett..

[55] Roccaro A.M., Sacco A., Maiso P., Azab A.K., Tai Y.T., Reagan M., Azab F., Flores L.M., Campigotto F., Weller E. (2013). BM mesenchymal stromal cell-derived exosomes facilitate multiple myeloma progression. Clin. Investig..

[56] Ji R., Zhang B., Zhang X., Xue J., Yuan X., Yan Y., Wang M., Zhu W., Qian H., Xu W. (2015). Exosomes derived from human mesenchymal stem cells confer drug resistance in gastric cancer. Cell Cycle.

[57] Del Fattore A., Luciano R., Saracino R., Battafarano G., Rizzo C., Pascucci L., Alessandri G., Pessina A., Perrotta A., Fierabracci A. (2015). Differential effects of extracellular vesicles secreted by mesenchymal stem cells from differential sources on glioblastoma cells. Expert Opin. Biol. Ther..

[58] Bailey A.J.M., Tieu A., Gupta M., Slobodian M., Schorr R., Ramsay T., Rodriguez R.A., Fergusson D.A., Lalu M.M., Allan D.S. (2021). Mesenchymal stromal cell-derived extracellular vesicles in preclinical animal models of tumor growth: Systematic review and meta-analysis. Stem Cell Rev. Rep..

[59] Vakhshiteh F., Atyabi F., Ostad S.N. (2019). Mesenchymal stem cell exosomes: A two edged sward in cancer therapy. Int. J. Nanomed..

[60] Ying H., Lin F., Ding R., Wang W., Hong W. (2020). Extracellular vesicles carrying MiR-193a derived from mesenchymal stem cells impede cell proliferation, migration and invasion in colon cancer by down-regulating FAK. Exp. Cell Res..

[61] Shang S., Wang J., Chen S., Tian R., Zeng H., Wang L., Xia M., Zhu H., Zuo C. (2019). Exosomal miRNA-1231 derived from bone marrow mesenchymal stem cells inhibits the activity of pancreatic cancer. Cancer Med..

[62] Katakowski M., Buller B., Zheng X., Lu Y., Rogers T., Osobamiro O., Shu W., Jiang F., Chopp M. (2013). Exosomes from marrow stromal cells expressing miR-146b inhibit glioma growth. Cancer Lett..

[63] Xu H., Zhao G., Zhang Y., Jing H., Wang W., Zhao D., Hong J., Yu H., Qi L. (2019). Mesenchymal stem cell-derived exosomal micro-RNA-133b suppresses glioma progression via Wnt/β-catenin signaling pathway by targeting EZH2. Stem Cell Res. Ther..

[64] Vakhshiteh F., Rahmani S., Ostad S.N., Madjd Z., Dinarvand R., Atyabi F. (2021). Exosomes derived from miR-34a-overexpressing mesenchymal stem cells inhibit in vitro tumor growth: A new approach for drug delivery. Life Sci..

[65] Luo G., Chen L., Xia C., Wang W., Qi J., Li A., Zhao L., Chen Z., Zheng M., Liu Y. (2020). MiR-199a-modified exosomes from adipose tissue-derived mesenchymal stem cells improve hepatocellular carcinoma chemosensitivity through mTOR pathway. J. Exp. Clin. Cancer Res..

[66] Quin F., Tang H., Zhang Y., Huang P., Zhu J. (2020). Bone marrow-derived mesenchymal stem cell-derived exosonal microRNA-208a promotes osteosarcoma cell proliferation, migration, and invasion. J. Cell Physiol..

[67] Ren W., Hou J., Yang C., Wang H., Wu S., Wu Y., Zhao X., Lu C. (2019). Extracellular vesicles secreted by hypoxia pre-challenged mesenchymal stem cells promote non-small cell lung cancer cell growth and mobility as well as macrophage M2 polarization via miR-21-5p delivery. J. Exp. Clin. Cancer Res..

[68] Guo Q., Yan J., Song T., Zhong C., Kuang J., Mo Y., Tan J., Li D., Sui Z., Cai K. (2021). MicroRNA-130b-3p contained in MSC-derived EVs promotes lung cancer progression by regulating the FOXO3/NFE2L2/TXNRD1 axis. Mol. Ther.-Oncolytics.

[69] He X., Smith S.E., Chen S., Li H., Wu D., Meneses-Giles P.I., Wang Y., Hembree M., Yi K., Zhao X. (2021). Tumor-intiating stem cell shapes its microenvironment into an immunosuppressive barrier and pro-tumorigenic niche. Cell Rep..

[70] Roda N., Blandano G., Pelicci P.D. (2021). Blood vessels and peripheral nerves as key player in cancer progression and therapy resistance. Cancers.

[71] Huang J., Zhang L., Wan D., Zhou L., Zheng S., Lin S., Qiao Y. (2021). Extracellular matrix and its therapeutic potential for cancer treatment. Signal Transduct. Target. Ther..

[72] Zhu Z., Parikh P., Zhao H., Givens N.T., Beck D.B., Willson C.M., Bai Q., Wakefield M.R., Peng Y. (2021). Targeting immune-metabolism of neoplasm by interleukin. A promising immunotherapeutic strategy for cancer. Cancer Lett..

[73] Bhat A.A., Nisar S., Maacha S., Carneiro-Lobo T.C., Akhtar S., Siveen K.S., Wani N.A., Rizwan A., Bagga P., Singh M. (2021). Cytokine-chemokine network driven metastasis in esophageal cancer; promising avenue for targeted therapy. Mol. Cancer.

[74] Vakhshiteh F., Zheng P., Li W. (2020). Crosstalk between mesenchymal stromal cells and tumor-associated macrophages in gastric cancer. Front. Oncol..

[75] Schroeder J.C., Puntigam L., Hofmann L., Jeske S.S., Beccard J.J., Doescher J., Laban S., Hoffmann T.K., Brunner C., Theodoraki M.-N. (2020). Circulating exosomes inhibit B cell proliferation and activity. Cancers.

[76] Yang Y., Li J., Geng Y. (2020). Exosomes derived from chronic lymphocyte leukemia cells transfer miR-146a to induce the transition from mesenchymal stromal cells into cancer-associated fibroblasts. J. Biochem..

[77] Olejarz W., Kubiak-Tomaszewska G., Chrzanowska A., Lorenc T. (2020). Exosomes in angiogenesis and anti-oncogenic therapy in cancer. Int. J. Mol. Sci..

[78] Zeng Y., Fu B.M. (2020). Resistance mechanisms of anti-angiogenic therapy and exosome-mediated revascularization in cancer. Front. Cell Dev. Biol..

[79] Atiya H., Frisbie L., Pressimone C., Coffman L. (2021). Mesenchymal stem cells in the tumor microenvironment. Adv. Exp. Med. Biol..

[80] Bai M., Li J., Yang H., Zhang H., Zhu Z., Deng T., Zhu K., Ning T., Fan Q., Ying G. (2019). miR-135b delivered by gastric tumor exosomes inhibits FOXO1 expression in endothelial cells and promotes angiogenesis. Mol. Ther..

[81] Yang Y., Lindsey-Boltz L.A., Vaughn C.M., Selby C.P., Cao X., Liu Z., Hsu D.S., Sancar A. (2021). Circadian clock, carcinogenesis chrono-chemotherapy connections. J. Biol. Chem..

[82] Xuan W., Khan F., James C.D., Heimberger A.B., Lenniak M.S., Chen P. (2021). Circadian regulation of cancer and tumor microenviranment crosstalk. Trends Cell Biol..

[83] Yoshida K., Yamamoto Y., Ochiya T. (2021). miRNA signaling network in cancer stem cells. Regen. Ther..

[84] Zhou X., Li T., Chen Y., Zhang N., Wang P., Liang Y., Long M., Liu H., Mao J., Liu Q. (2019). Mesenchymal stem cell-derived extracellular vesicles promote the in vitro proliferation and migration of breast cancer cells through the activation of the ERK pathway. Int. J. Oncol..

[85] Shen Y.A., Chen C.C., Chen B.J., Wu Y.T., Juan J.R., Chen L.Y., Teng Y.C., Wei Y.H. (2021). Potential therapies targeting metabolic pathways in cancer stem cells. Cells.

[86] Tieu A., Lalu M.M., Slobodian M., Gnyra C., Fergussson D.A., Montroy J., Burger D., Stewart D.J., Allan D.S. (2020). An analysis of mesenchymal stem cell-derived extracellular vesicles for preclinical use. ACS Nano.

[87] Seo Y., Kim H.S., Hong I.S. (2019). Stem cell-derived extracellular vesicles as immunomodulatory therapeutics. Stem Cells Int..

[88] Pinto A., Marengon I., Meréaux J., Nicolàs-Boluda A., Lavieu G., Wilhelm C., Sarda-Mantel L., Silva A.K.A., Pocard M., Gazeau F. (2021). Immune reprogramming precision photodynamic therapy of peritoneal metastasis of scalable stem cell-derived extracellular vesicles. ACS Nano.

[89] Yokoe I., Omata D., Unga J., Suzuki R., Maruyama K., Okamoto Y., Osaki T. (2021). Lipid bubbles combined with low-intensity ultrasound enhance the intratumoral accumulation and antitumor effect of pegylated liposomal doxorubicin in vivo. Drug Deliv..

[90] Bageri E., Abnous K., Ferzad S.A., Taghdisi M., Ramezani M., Alibolandi M. (2020). Targeted doxorubicin-loaded mesenchymal stem cell-derived exosomes as a versatile platform for fighting against cholorectal cancer. Life Sci..

[91] Wiest E.F., Zubair A.C. (2020). Challenges of manufacturing mesenchymal stromal cell-derived extracellular vesicles in regenerative medicine. Cytotherapy.

[92] Xie F.Y., Xu W.H., Yin C., Zhang G.Q., Zhong Y.Q., Gao J. (2016). Nanomedicine strategies for sustained, controlled and targeted treatment of cancer stem cells of digestive system. World J. Gastrointest. Oncol..

[93] Zhao Q., Hai B., Kelly J., Wu S., Liu F. (2021). Extracellular vesicle mimics made from iPS cell-derived mesenchymal stem cells improve the treatment of metastatic prostate cancer. Stem Cell Res. Ther..

[94] Hayat H., Hayat H., Dwan B.F., Gudi M., Bishop J.O., Wang P. (2021). A concise review: The role of stem cells in cancer. Onco Targets Ther..

[95] Hervieu C., Christou N., Battu S., Mathonnet M. (2021). The role of cancer stem cells in colorectal cancer: From the basic to novel clinical trials. Cancers.

[96] Gao H.F., Lin Y.Y., Zhu T., Zhang L.L., Yang C.Q., Yang M., Li J.Q., Ceng M.Y., Wang K. (2021). Adjuvant inhibitors combined with endocrine therapy in HR-positive HER2-negative early breast cancer: A meta-analysis of randomized clinical trials. Breast.

[97] Hernandez-Oller L., Seras-Franzoso J., Andrade F., Rafael D., Abasolo I., Gener P., Schwartz S. (2020). Extracellular vesicles as drug delivery system in cancer. Pharmaceutics.

[98] Riazifar M., Pone E.J., Lotvall J., Zhao W. (2017). Stem cell extracellular vesicles: Extended messages and regeneration. Ann. Rev. Pharmacol. Toxicol..

[99] Movahed Z.G., Yarami R., Mohammadi P., Mansouri K. (2021). Sustained oxidative stress instigates differentiation of cancer stem cells into tumor endothelial cells: Pentose phosphate pathway, reactive oxygen species and autophagy crosstalk. Biomed. Pharmacother..

[100] Patsalias A., Kozovska Z. (2021). Personalized medicine: Stem cells in colorectal cancer treatment. Biomed. Pharmacother..

[101] Hassanzadek A., Rahman H.S., Markov A., Endiun J.J., Zekiv A.O., Chardrand S., Beheshtkoo N., Kouhbanani A.J., Marofi F., Nikoo M. (2021). Mesenchymal stem/stromal cell-derived exosomes in regenerative medicine and cancer; overview of development, challenges, and opportunities. Stem Cell Res. Ther..

[102] Trosko J.E. (2005). The role of stem cells and gap junctions as targets for cancer prevention and chemotherapy. Biomed. Pharmacother..

[103] Van Niekerk G., Davids L.M., Hattingh S.M., Engebrecht A.M. (2017). Cancer stem cells: A product of clonal evolution?. Int. J. Cancer.

[104] Kaushik V., Kukkarni Y., Felix K., Azad N., Iver A.K.V., Yakisich J.S. (2021). Alternative models of cancer stem cells: The stemness phenotype model, 10 years later. World J. Stem Cells.

